# The complete genome sequence of a *Paenibacillus tundrae* strain MLY93 isolated from spot disease-infected tobacco leaves in China

**DOI:** 10.1128/mra.00221-24

**Published:** 2024-09-26

**Authors:** Guan Lin, Xianhai Yang, Dong Xiang, Lei Yang, Yong Liu, Huilin Zhang, Shiwang Liu

**Affiliations:** 1 School of Biological & Chemical Engineering, Zhejiang University of Science & Technology, Hangzhou, China; 2 China Tobacco Hunan Industrial Co. Ltd., Changsha, China; University of Strathclyde, Glasgow, United Kingdom

**Keywords:** complete genome, *Paenibacillus tundrae*, tobacco spot disease

## Abstract

This study presents the complete gene sequence of a *Paenibacillus tundrae* strain isolated from tobacco spot disease leaves in Xingyi, Guizhou Province, China. The genetic understanding of *P. tundrae* is advanced by this research.

## ANNOUNCEMENT

*Paenibacillus tundrae* belongs to the genus *Paenibacillus,* which was first separated from *Bacillus* in 1994 (1). *P. tundrae* is characterized as a gram-positive, rod-shaped organism surrounded by flagella and can grow under aerobic or anaerobic conditions (2, 3). Rasimus isolated *P. tundrae* from barley grain; it could produce cereulide-like depsipeptides that are highly toxic to mammalian cells (4). Here, we present the whole-genome sequence of the *P. tundrae* strain.

The study utilized biological samples isolated from a leaf belonging to *Nicotiana tabacum* affected by tobacco spot disease in Xingyi, Guizhou Province, China (25.08 N 104.90 E). Diseased leaf samples were first sterilized with 75% ethanol to disinfect the surface of leaf veins, after which the vein surface was removed. 0.5 g of leaf vein tissue was ground and mixed well in 10 mL of sterile water, and this solution was diluted 100-fold, and 100 µL was aspirated and inoculated into NA medium (1 L of medium contained 10 g peptone, 2 g yeast extract, 6 g beef extract, 10 g sucrose, 5 g sodium chloride, and 15 g Agar). After incubation for 48 h at 30°C, re-streaking of single colonies was performed three successive times to purify the strain MLY93. DNA was extracted using MGIEasy Microbiome DNA Extraction Kit (MGI Tech, China) after incubation for 48 h in NB liquid medium at 30°C and 170 rpm/min.

The DNA fragments were sheared to 15–20 Kb using Megaruptor on the PacBio RSII platform. SMRTbell were utilized for end-to-end connection of DNA fragments, forming a dumbbell-shaped structure, followed by size selection and accurate recovery with Sage ELF to obtain libraries with very high fragment size consistency. Raw sequencing data were filtered out adapter sequences and fragments less than 1,000 bp in length to obtain Subreads (2,390,162 Subreads with an average length of 11,414 bp and an N50 value of 12,028 bp). Circular Consensus Sequencing Subreads were obtained using SMRT Analysis v2.3.0 (5) and self-corrected and assembled using Canu v1.5 (6). A single contig was assembled from the subreads. Single base correction of the assembly was performed using GATK v3.4-0-g7e26428 (7) according to subreads. Circularization determination was achieved by comparing whether there was overlapping sequence between the start and end, using the software Blast v2.2.30+. If the overlap between the first and last sequences was greater than 5K and the similarity was greater than 0.95, the overlap was considered. The genome was annotated with PGAP v6.6 (8). Average Nucleotide Identity (ANI) was calculated using fastANI v1.32 (9). Genome circle diagrams were plotted using Circos v0.69-6 (10). Note that the default parameters were used except where otherwise noted.

**Fig 1:**
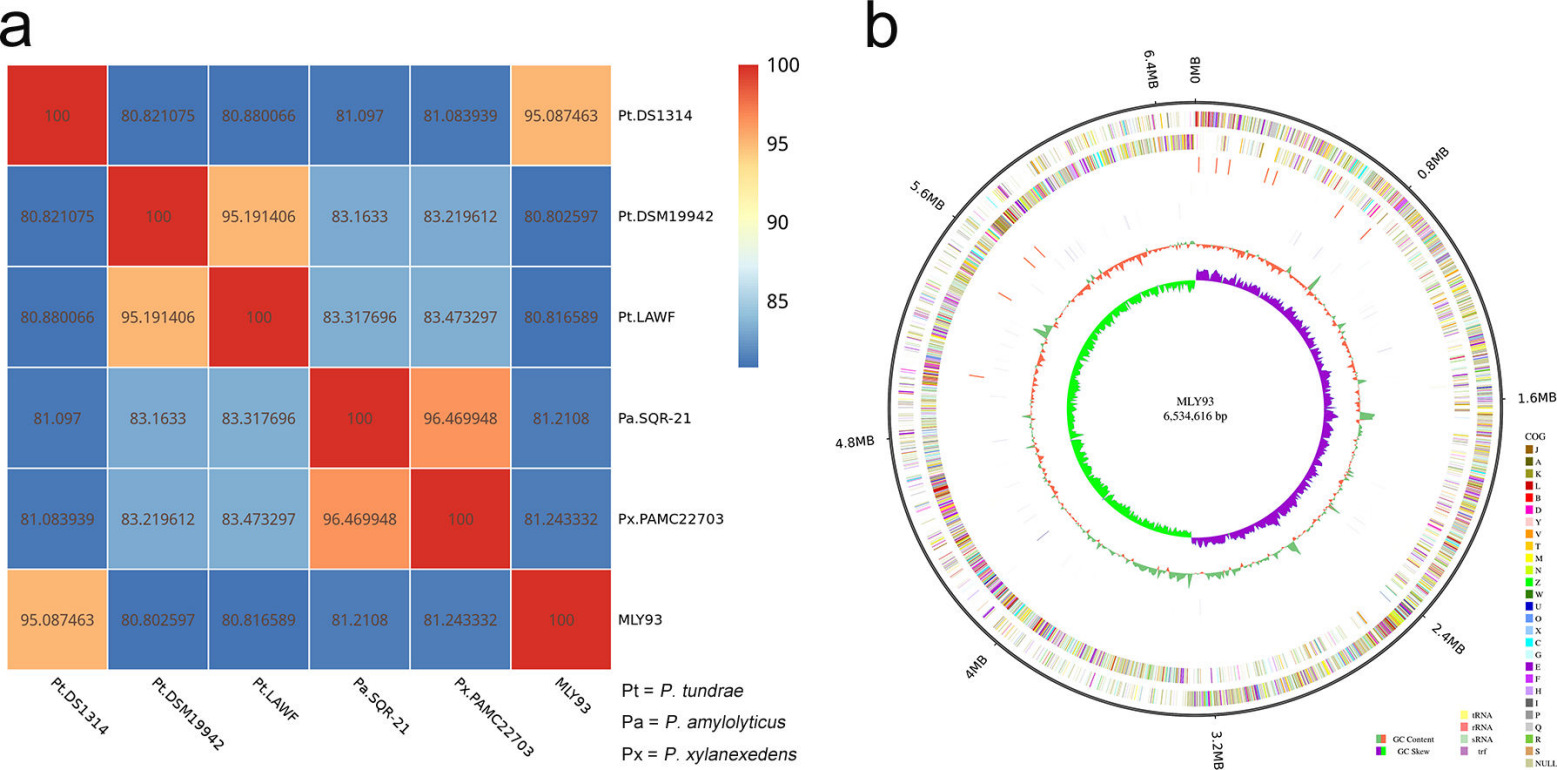
(**A**) ANI Heat map; (**B**) Circle map of the MLY93 genome.

Table 1 displays the genome characterization of this strain. Strain MLY93 belongs to the genus *Paenibacillus*, and the ANI value between its genome and the publicly available *P. tundrae* (DS1314, accession number JAUSTI010000000) was calculated to be 95.09% (Fig. 1A). Fig. 1B shows the genome circle diagram based on gene distribution, ncRNA distribution, annotation, and other data.

**TABLE 1:** Genome features of MLY93

Genetic element	Number
Genome size (bp)	6,534,616
G + C content (%)	44.77
Number of genes	5,828
Genome coverage	40.97
tRNAs	101
sRNAs	7
Length of 16S rRNA (bp)	1,541
Accession numbers	SRR26804258

## Data Availability

The raw reads for genome sequencing have been deposited in the NCBI Sequence Read Archive under accession numbers SRR26804258. The MLY93 genome sequence was deposited in the DDBJ/ENA/GenBank database under accession number CP145605.1 (BioProject/BioSample PRJNA1039734/SAMN38224731).
